# Evidence-based Risk Stratification for Sport Medicine Procedures During the COVID-19 Pandemic

**DOI:** 10.5435/JAAOSGlobal-D-20-00083

**Published:** 2020-10-01

**Authors:** Betina B. Hinckel, Charles A. Baumann, Leandro Ejnisman, Leonardo M. Cavinatto, Alexander Martusiewicz, Miho J. Tanaka, Marc Tompkins, Seth L. Sherman, Jorge A. Chahla, Rachel Frank, Guilherme L. Yamamoto, James Bicos, Liza Arendt, Donald Fithian, Jack Farr

**Affiliations:** From the Oakland University, Rochester (Dr. Hinckel, and Dr. Cavinatto); Department of Orthopaedic Surgery, William Beaumont Hospital, Royal Oak (Dr. Hinckel, Dr. Cavinatto), MI; the University of Missouri—School of Medicine, Columbia, MO (Mr. Baumann); the Hospital das Clínicas HCFMUSP, Faculdade de Medicina, Universidade de São Paulo, Sao Paulo, SP, BR (Dr. Ejnisman); the Shoulder and Elbow Surgery, Beaumont Orthopaedic Associates, Beaumont Health (Dr. Martusiewicz); the Department of Orthopaedic Surgery, Massachusetts General Hospital, Harvard Medical School, Boston, MA (Dr. Tanaka); the Department of Orthopedic Surgery, TRIA Orthopedic Center, University of Minnesota, Gillette Children's Specialty Healthcare, MN (Dr. Tompkins); the Department of Orthopedic Surgery, Stanford University, CA (Dr. Sherman); the Rush University Medical Center, Chicago, IL (Dr. Chahla); the Division of Sports Medicine and Shoulder Surgery, Department of Orthopedics, Aurora, CO (Dr. Frank); the Department of Orthopaedic Surgery, Boston Children's Hospital, Harvard Medical School, Boston, MA (Dr. Yamamoto); CEGH-CEL, Instituto de Biociências, Universidade de São Paulo (Dr. Yamamoto); DASA Laboratories, Sao Paulo, Brazil (Dr. Yamamoto); the Michigan Orthopedic Surgeons, Fellowship Director William Beaumont Sports Medicine Fellowship, Assistant Professor Oakland University William Beaumont School of Medicine, MI (Dr. Bicos); the Department of Orthopaedic Surgery, University of Minnesota, Minneapolis, MN (Dr. Arendt); the Southern California Permanente Medical Group and Torrey Pines Orthopaedic Medical Group, San Diego, CA (Dr. Fithian); and the Knee Preservation, Cartilage Regeneration and OrthoBiologics, Department of Orthopedic Surgery, Indiana University School of Medicine, OrthoIndy and OrthoIndy Hospital, Greenwood and Indianapolis, IN (Dr. Farr).

## Abstract

Orthopaedic practices have been markedly affected by the emergence of the COVID-19 pandemic. Despite the ban on elective procedures, it is impossible to define the medical urgency of a case solely on whether a case is on an elective surgery schedule. Orthopaedic surgical procedures should consider COVID-19-associated risks and an assimilation of all available disease dependent, disease independent, and logistical information that is tailored to each patient, institution, and region. Using an evidence-based risk stratification of clinical urgency, we provide a framework for prioritization of orthopaedic sport medicine procedures that encompasses such factors. This can be used to facilitate the risk-benefit assessment of the timing and setting of a procedure during the COVID-19 pandemic.

Globally, as of May 2, 2020, there were 3,233,191 confirmed cases of COVID-19 with 227,489 associated deaths.^1^ In the Unites States alone, there were 1,067,127 confirmed cases of Coronavirus disease 2019 (COVID-19) with 57,406 deaths.^1^

Few states have published guidelines specific to orthopaedic surgery during the COVID-19 outbreak, leaving hospital systems and surgeons with the responsibility of balancing the benefits of surgery with the risks to public health.^2^ Before March 24, 30 states published guidance regarding the discontinuation of elective procedures and 16 states provided a definition of “elective” procedures or specific guidance for determining which procedures should continue to be performed. Only five states provided guidelines specifically mentioning orthopaedic surgery; of those, four states explicitly allowed for trauma-related procedures and four states provided guidance against performing arthroplasty.^2^ On April 16, 2020, The White House released a three-phased guideline, called Opening Up America Again, for state and local authorities to follow when reopening their economies. In phase 1, for states and regions that satisfy the gating criteria, “elective surgeries” can resume when appropriate and on an outpatient basis.^3^

Mi et al^4^ showed that clinical characteristics and early prognosis of COVID-19 in patients with fractures were more severe than those reported for adult patients with COVID-19 without fractures, suggesting that fractures can worsen the course of the infection. Catellani et al^5^ concluded that surgical treatment of femoral fragility fractures in COVID-19-positive patients not only contributed to the overall patients' mobility but also improved the physiologic ventilation, O^2^ saturation, and assisted respiration, indicating that appropriate treatment improves the patients' overall clinical status. Dephillipo et al^6^ reported on acute orthopaedic injuries they recommend as “surgically necessary” for elective-urgent procedures at Ambulatory Surgical Centers (ASCs); however, they did not provide literature support to these recommendations, nor did they identify the timeframe in which surgeries should be performed. Therefore, an evidence-based risk stratification for orthopaedic pathologies has yet to be established. In addition, in the context of a pandemic, it is important to integrate the disease-intrinsic factors to external factors (eg, epidemic situation, healthcare system situation, and patient characteristics) to decide whether time-sensitive surgeries should be performed in a specific patient in a particular scenario.

The purpose of this manuscript is to provide a clear framework for the prioritization of orthopaedic sport medicine procedures. This evidence-based risk stratification based on clinical urgency facilitates the risk-benefit assessment of whether, when, and in what setting, surgery should be performed during the COVID-19 pandemic. The authors discuss all phases of the pandemic, the initiation, and acceleration intervals, as well as the deceleration intervals, when elective surgeries are gradually allowed back. A framework for prioritization, such as the one presented in this study, is recommended by the American College of Surgeons, and it will continue to serve as an important guide, both during and after the pandemic.

## Risk Stratification

We synthesized the current knowledge of common orthopaedic sport-medicine ailments from published literature and expert opinion to develop consensus statements and tables for each topic. For each condition, the assessment tables contain coded cells, with green boxes representing favorable situations to perform surgery, yellow moderate, and red unfavorable.

### Disease Independent or External Factors Risk Assessment

Ultimately, disease independent or “external” factors must determine the safe resumption of nonemergent orthopaedic surgery. The changes in risk are a continuum, and we must take that into consideration when stratifying those parameters and adapting them to categorical variables. Therefore, they should be evaluated on a case-by-case basis and seen as relative considerations and not absolute, especially when it comes to patient risk and transmission risk because that are rapidly evolving areas of knowledge. Table 1 presents a suggested guideline for risk stratification based on external factors. Factors included in disease-independent factors are as follows:

1. Status of the epidemic. This depends on the local infection rate and containment. This is determined by federal and local authorities as well as the Centers for Disease Control and Prevention (CDC).
2. Hospital capacity. This relates to the hospital availability of resources (eg, equipment and personal) to care for patients with COVID-19 and other patients during the current time point and future projections, the expected progression of pandemic. This is determined by the local health systems and authorities that collect and provide resources as well as facilitate integration between different systems to distribute patient load.
3. ASCs availability. Theoretically, ASC settings not associated with active care of sick patients are a safer setting for patients. It is important to remember that personal protective equipment are a common good shared across the whole healthcare system (including hospitals and urgent cares); therefore, the availability of those personal protective equipment depends on the demands of centers taking care of patients with COVID-19.
4. Patient risk for COVID-19 complications. Older patients and patients with comorbidities are at higher risk for developing acute respiratory distress syndrome, need for intensive care unit (ICU) admission, and death. Therefore, they are at increased risk when entering healthcare facilities, more so in hospitals than ASCs, and during emergency states of the epidemic. In addition, if they develop complications, they further overload hospital resources. Also, patients who are already infected have increased risk of complications compared with noninfected peers.
5. ASA physical status classification system. Patients with comorbidities are at higher risk for developing acute respiratory distress syndrome, need for ICU admission, and death. In addition, they might need longer hospital stay (which further increases the risk of COVID-19 and non-COVID-19 complications) and uses more hospital resources that might be in a nonideal situation.
6. Transmission risk of COVID-19 assessment because of patient status. Patients who are known to be infected with COVID-19 are more likely to transmit the disease to healthcare providers (HCPs) and other patients, especially if they are symptomatic, and more so in severe forms of disease. For patients with unknown status because of lack of testing, the risk is influenced by the situation of the epidemic and close contacts. Surgeons can consider testing to better evaluate the risk, understanding that false positive and false negative exists.

**Table 1:** Disease-independent Risk Assessment Table for Assisting in Surgical Decision-making

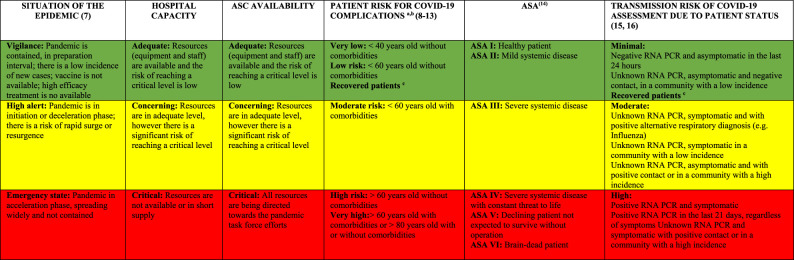

ASC = ambulatory surgical center, ASA = American Society of Anesthesiologists, IgG = immunoglobulin G, PCR = polymerase chain reaction

a Patients already infected with COVID-19 have a higher risk when compared with noninfected peers.

b Decreases when the situation of the epidemic and the hospital capacity improve to green.

c Previously positive RNA PCR, currently symptomatic, 14 days after IgG positive, or those with resolved symptoms that started >21 days ago.

### Disease-Specific Risk Assessment

Tables 2 contains the disease-specific risk-benefit assessment. For each pathology/procedure, the authors provide the following regarding the frequency and utilization of resources for each case: the incidence, number of surgeons/assistants, anesthesia methods, surgical time, cost, short- and long-term disability, cost-effectiveness, risk for COVID-19 complications, risk for surgical complications, postsurgical needs for social/home support. Appendix 1, http://links.lww.com/JG9/A84, contains a detailed review regarding disease-specific risk assessment.

**Table 2:** Disease-Specific Risk Assessment Table for Assisting in Surgical Decision-making

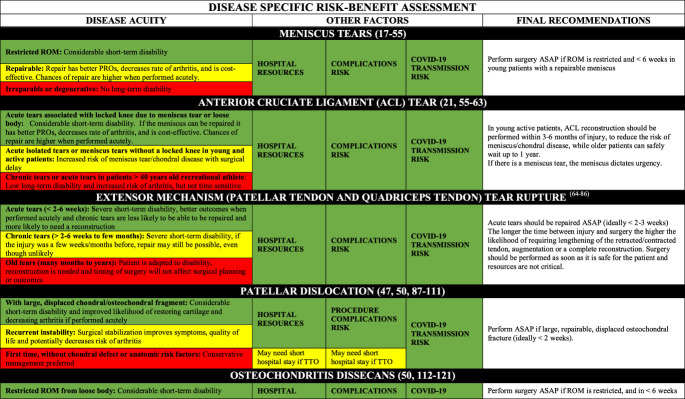 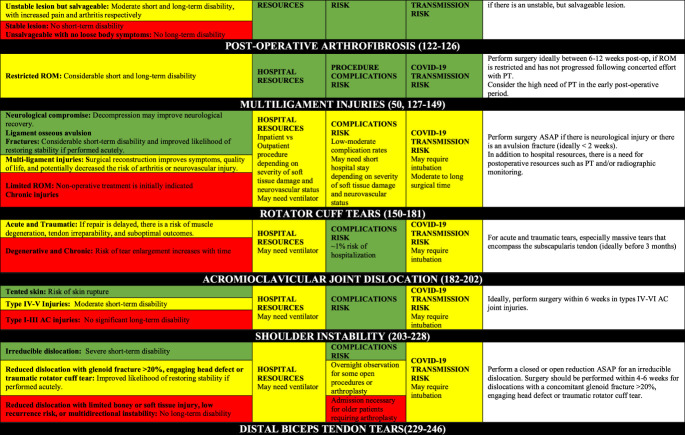 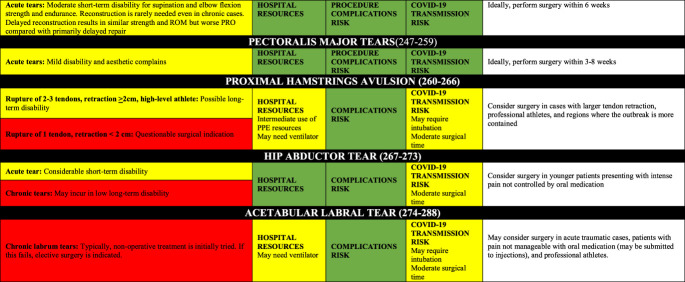

AC = acromioclavicular, ACL = anterior cruciate ligament, ASAP = as soon as possible, PPE = personal protective equipment, PROs = patient-reported outcomes, PT = physical therapy, ROM = range of motion

In addition, the ideal timing for surgery depends on short-term and long-term outcomes, as well as time sensitivity. Of note, limb-threatening (eg, vascular compromise and compartment syndrome) and life-threatening (eg, open fractures and polytrauma) conditions are emergencies and are not included here. The nonemergent surgeries can be classified as the following:

1. Urgent surgery (green boxes): Strong evidence that any delay will result in inferior outcomes, or strong consensus that surgery within weeks is necessary for acceptable outcomes. Should be performed as soon as possible (a few days to a few weeks).
2. Time-sensitive surgery (yellow boxes): Moderate to strong evidence that delayed surgery contributes to inferior surgical outcomes (should be performed in a few weeks to a few months).
3. Not time-sensitive/elective surgery (red boxes): Absence of moderate to strong evidence of a notable relationship between surgical timing and outcomes, and the absence of consensus on delayed surgery on outcomes. They can be postponed a few months without major ramifications to the patient other than a lengthier time dealing with pain and life limitations/restrictions; however, it is important to highlight that they are necessary to improve patients' symptoms and quality of life and should not be postponed indefinitely.

Hospital resource use, procedure complications risk, and transmission of COVID-19 risk because the procedure for each procedure were summarized in Table 2.

In combining Tables 1 and 2, one can have a comprehensive understanding of the risks and benefits of proceeding with surgery. In addition, there are many important considerations orthopaedic surgeons must consider during the COVID-19 pandemic. We urge that those “low-risk geographic areas” to exercise caution because some states have reported few cases, but this may be because of slow pace of testing, where many more people are believed to be infected. There is a higher patient risk and associated liability in performing surgery in an “emergency state” area. In these areas, particularly if the hospital capacity is critical, only perform surgeries that are urgent (green boxed) and have other favorable green boxes.

#### Patient-Related Factors

If a patient has COVID-19, consider postponing surgery, decreasing the patient's risk of complications (surgery suppresses the immune system) and transmission to healthcare providers (HCPs) and other patients. Procedures and operations should be performed if delaying the procedure or operation is likely to prolong the patient's hospital stay while waiting for the surgery, increasing the likelihood of later hospital admission or causing harm, all increasing the risk of the patient acquiring COVID-19. In patients who are suspected to be infected with COVID-19 (eg, have typical symptoms, have close contact with infected persons, or live in a community with high incidence), consider testing RNA polymerase chain reaction (PCR) testing. This not only benefits the patient but also improves the hospital management/operations. For “gray area cases” (mostly yellow boxes), do no harm by assessing the patient's risk with your own judgement. No substitute exists for sound surgical judgement. For elective surgery (red-boxed diseases), surgery should wait until most other categories have normalized (green boxes). In addition, consideration should be given to the fact that there may be a large need for physical therapy postoperatively for an optimal result, which may not be available during certain phases of the pandemic.

#### Hospital Setting

If the patient needs to stay in the hospital, especially for many days, hospital capacity is more relevant than it is for outpatient surgery, for which the status of ASCs is more important. Inpatient procedures not only take on a bed that may be a scarce resource but it also places the patient at a higher risk for acquiring COVID-19. ASCs should have arrangements with a hospital if overnight stay for outpatient surgery becomes necessary because surgeons try to bring more patients to ASCs. In addition, it needs to be taken into consideration if it is anticipated that after surgery, the patient will need other resources, such as intensive care unit (ICU) bed and blood products.

#### Proceeding With Surgeries

Once the surgeon decides to proceed with surgery, all appropriate precautions should be taken. The CDC infection control guidelines can be found at https://www.cdc.gov/coronavirus/2019-ncov/hcp/infection-control.html. The American College of Surgeons offers guidance on considerations for optimal surgical protection before, during, and after operations. For full guidelines, go to https://www.facs.org//media/files/covid19/considerations_optimum_surgeon_protection.ashx.

The evidence-based risk stratification presented in this study has the limitation of not being clinically validated, similar to the guidelines from the World Health Organization, CDC, or other institutions. However, we think we provide a comprehensive review of the literature that is optimal and the best available, considering the urgency and complexity of these times and the limited literature regarding this topic.

## Conclusion

The medical urgency of a case cannot be defined solely on whether a case is on an elective surgery schedule. Plans for orthopaedic case triage should avoid blanket policies and instead depend on disease-specific data and expert opinion from qualified orthopaedic surgeons. Although COVID-19 is a risk to all, it is one of several competing risks for patients with functional limitations necessitating orthopaedic surgical care. Therefore, we provide guidelines based on an assimilation of all available disease-dependent, disease-independent, and logistical information to help guide surgeons and institutions in the decision-making process.
